# *Withania somnifera* (L.) Dunal whole-plant extract demonstrates acceptable non-clinical safety in rat 28-day subacute toxicity evaluation under GLP-compliance

**DOI:** 10.1038/s41598-022-14944-x

**Published:** 2022-06-30

**Authors:** Acharya Balkrishna, Sandeep Sinha, Jyotish Srivastava, Anurag Varshney

**Affiliations:** 1 Drug Discovery and Development Division, Patanjali Research Institute, NH-58, Roorkee-Haridwar Road, Haridwar, Uttarakhand 249 405 India; 2 Department of Allied and Applied Sciences, University of Patanjali, NH-58, Haridwar, Uttarakhand 249405 India; 3 Patanjali UK Trust, Glasgow, UK; 4 Special Centre for Systems Medicine, Jawaharlal Nehru University, New Delhi, 110067 India

**Keywords:** Drug discovery, Drug safety

## Abstract

*Withania somnifera* (L.) Dunal (Ashwagandha) is widely used in Ayurveda, Unani and Siddha systems of medicines due to its therapeutic application in numerous ailments. Traditionally, the medications prepared from the plant employ only its roots and based on the currently available scientific literature, their efficacy and safety is well established. Apart from the roots, the aerial parts also contain bioactive components and correspondingly certain marketed preparations also employ the leaves of the plant. Accordingly, Ministry of Ayush, Government of India has lately issued an advisory emphasizing the need for extensive efficacy and safety profiling of leaf-based products. Consequently, we have conducted the present GLP-driven study, in which the non-clinical safety of a hydromethanolic extract of the whole plant of *Withania somnifera* (WSWPE) has been assessed according to OECD guideline 407. In this study Sprague Dawley rats of either sex were orally administered with WSWPE for 28-consecutive days at the doses of 100, 300 and 1000 mg/kg/day. The study also included a satellite group of animals that received WSWPE for 28-days followed by a 14-days recovery period. *Withania somnifera* Whole Plant Extract was found to be safe up to the dose level of 1000 mg/kg/day as no toxicologically relevant findings could be detected.

## Introduction

*Withania somnifera* (L.) Dunal (Hindi: Ashwagandha, English: Winter Cherry), belonging to the family Solanaceae, has been employed in Ayurveda for the treatment of wide array of diseases. Historically, its presence as a standard therapeutic herb, in the Indian systems of medicines, dates back to thousands of years^1^. Traditionally, the dried roots of the herb are used to prepare classical Ayurvedic formulations and modern science has also validated its beneficial effects through evidence-based research, centered on deciphering its pharmacological mechanism of action. The pharmacological activity of *Withania somnifera* is attributed to the presence of several bioactive secondary metabolites present in the roots^2^. In preclinical studies, a wide variety of extracts prepared from the roots have demonstrated anti-cancer, immunomodulatory, cardioprotective, neuroprotective, anti-oxidant, anti-ageing, adaptogenic and anti-diabetic activities^3^. With respect to its neuroprotective activity, oral administration of *Withania somnifera*, in an animal model of Parkinson’s disease, improved the striatal catecholamine contents and upregulated the dopamine D2 receptors in the striatum^2^. These biological activities have fruitfully translated into demonstrable clinical efficacies in a range of disorders such as hypothyroidism, schizophrenia, obsessive compulsive disorder, stress, anxiety, insomnia, cognitive impairment, Type 2 diabetes mellitus and male infertility^3^.

Whilst dried roots or extracts are chiefly utilized for their therapeutic use, certain over-the-counter formulations additionally employ leaf-based formulations. Regarding the usage of such marketed products, an advisory has recently been issued by the Drug Policy Section, Ministry of AYUSH, Government of India, which asserts that currently there is an impending need for substantial evidence and scientific literature, which can unequivocally establish the efficacy and safety of formulations prepared from the leaves of *Withania somnifera*^4^. Generation of rigorous and high quality data would subsequently pave the way for the regulatory acceptance of the formulations obtained from the leaves, thereby enabling their therapeutic use without any reservation. Accordingly, with these well-defined directives from the regulatory agencies in mind, we report for the first time a GLP-directed non-clinical safety assessment of an extract prepared from the whole plant of *Withania somnifera*, which include the leaves and the other aerial parts.

It is imperative to comprehend that apart from the roots, the extracts prepared from leaves and stem^5^, berries^6^ and the seeds of *Withania somnifera*^7^ contain several bioactive components, which have, in addition to the roots demonstrated preclinical pharmacological activities with potential clinicotherapeutic applications. Therefore, the aerial parts of the plant also merit further clinical investigation, in addition to the roots.

The preclinical and clinical safety of the root preparations have been extensively studied. The non-clinical safety evaluation studies have revealed an acceptable safety profile of *Withania somnifera* root. Various acute, subacute, sub-chronic and chronic toxicity studies have effectively ascertained that the root extracts are safe in experimental animals upon oral administration, which is the intended route in human subjects^1^. Likewise, there is sufficient evidence of an adequate clinical safety of the root preparations in published literature and no serious safety concerns including alteration in vital signs, hematological and clinical chemistry parameters have been reported till date. In clinical trials, all the reported adverse events range from mild to moderate and are of a transient nature and withdrawal from trials on account of adverse events is reported to be negligible^1^.

In our view, owing to the similarity of the bioactive secondary metabolites present in the roots as well as the aerial parts of the plant, we expect a comparable safety profile between the root and the whole plant extract. Hence, with an aim to support the long term safety evaluation in rodents, safety assessment in non-rodents and the prospective clinical utility of *Withania somnifera* Whole Plant Extract (WSWPE), we have determined its non-clinical safety in accordance with Organization for Economic Cooperation and Development (OECD) guideline 407^8^ and in conformance with OECD Principles of Good Laboratory Practice (GLP). In the present study, WSWPE was repeatedly administered by oral route to Sprague Dawley rats of either sex for a total duration of 28 days. Our study design has also incorporated a satellite arm of animals that were orally administered with the highest dose of the extract for 28 days and were then observed for an additional 14 treatment-free days to evaluate the persistence, reversibility or the delayed manifestation of adverse effects. The main objective of the study was to furnish evidence of any extract-associated major toxicity along with the target organs involved and the No-Observed-Adverse-Effect-Level (NOAEL) of WSWPE in rats. In our knowledge this is the first report describing the non-clinical safety evaluation of the whole plant extract of *Withania somnifera*.

## Methods

### Test item, chemicals and reagents

The whole plant of *Withania somnifera* (L.) Dunal was collected from the Herbal Garden of Patanjali Research Institute, Haridwar, India and it comprised of the roots, stem, leaves, berries and seeds in natural ratio *Withania somnifera* whole plant was collected under compliance with the institutional, national, and international guidelines. The authenticity of the plant sample was certified by the Council of Scientific and Industrial Research–National Institute of Science Communication and Information Resources (CSIR–NISCAIR), New Delhi, Government of India vide authentication voucher number NISCAIR/RHMD/Consult/2019/3453-54-15; and Patanjali Research Foundation Herbarium (vide accession number 5606). WSWPE (Batch number D4/CHM/SAHA075/1220), a brown colored, free-flowing powder, was generated at Patanjali Research Institute, Haridwar India. An aqueous suspension of WSWPE, intended for oral administration to the experimental animals, was formulated by employing 0.5% methylcellulose as the vehicle. All of the other reagents and chemicals utilized for the experiment were of the highest commercial quality.

### Preparation of WSWPE

About 10 kg of dry *Withania somnifera* whole plant material was extracted with 75 L of Water: Methanol (1:1 v/v) as the solvent at 70–80 °C for 4 h in an extractor under reflux condition. The extract was filtered through a 10-micron filter cloth. This process was repeated once again to assure complete extraction. The filtrate was pooled and concentrated in wiped film evaporator and spray dried with inlet air temperature of 150 °C and feed flow rate of 10 L per hour. A light to dark brown colored powder was obtained with a yield of 11% on dry basis.

### Compositional and stability analysis of WSWPE suspension

WSWPE suspension of concentration 100 mg/mL in 0.5% methylcellulose was analyzed for its constituents, immediately after preparation and after retaining it for 24 h at room temperature. For the preparation of the test solution, 10 mL of methanol was added to 1.0 gm of the suspension. The solution was then centrifuged for 5 min at 5000 rpm and filtered using a 0.44 μm nylon filter. The standards employed included Withanoside IV and Withanolide A, sourced from Natural Remedies, India. Stock solutions of both the standards (Concentration: 1000 µg/mL) were prepared by dissolving accurately weighed standards in methanol. The stock solutions of the standard were further diluted to prepare 100 µg/mL solutions. Ultra high performance chromatography (UHPLC) analysis was performed on Prominence-XR UHPLC system (Shimadzu, Japan) equipped with a photo diode array detector. A gradient solvent system of solvent A: prepared by dissolving 0.14 gm potassium dihydrogen phosphate in 900 mL of water with the addition of 0.5 mL orthophosphoric acid and then diluted to 1000 mL with water; and solvent B: acetonitrile was used. Gradient elution program used during the analysis consisted of 5 to 45% B for 0 to 18 min, 45 to 80% B from 18 to 25 min, 80% B from 25–28 min, 80 to 5% B from 28 to 30 min, 5% B from 30 to 40 min. The Shodex C18 4E (5 µm, 4.6 × 250 mm) (Shodex, Japan) with a flow rate of 1.5 mL/min was used for chromatographic separation. The chromatographic detection of all the analytes was performed using a PDA detector at 227 nm. The temperature of the column was kept at 27 °C and the sample injection volume was 20 µL.

Identification of analytes were done on the basis of relative retention time (RRT) where the Withanolide A peak was used as a reference and consider as 1.0 RRT whereas 0.70, 0.75, 0.80, 0.89, 0.89, 0.92, 0.96, 1.01 and 1.14 were the RRT of Withanoside IV, Physagulin D, 27-hydroxywithanone, Withanoside V, Withaferin A, 12-deoxywithastramonolide, Withanone and Withanolide B, respectively.

The concentration of the individual analytes in the suspension of WSWPE was calculated as per their category that is Withanolide aglycones and Withanolide glycosides. Withanolide aglycones including Withaferin A, 12-deoxywithastramonolide, Withanolide A, Withanone and Withanolide B, were calculated against Withanolide A standard sample whereas Withanolide glycosides (Withanoside IV, Withanoside V and Withanoside VI) were calculated against Withanoside IV standard sample.

### Non-clinical safety assessment test facility

The non-clinical safety assessment of WSWPE was conducted at Preclinical Research and Development Organization (PRADO), Private Limited, Pune, India. The test facility is certified by National GLP Compliance Monitoring Authority (NGCMA), Department of Science & Technology, Government of India (GLP/C-127/2018; GLP/C-168/2021); and by the Committee for the Purpose of Control and Supervision of Experiments on Animals (CPCSEA; Registration number: 1723/PO/RcBiBt/S/13/CPCSEA), Department of Animal Husbandry and Dairying, Ministry of Fisheries, Animal Husbandry and Dairying, Government of India. Coded test item was supplied to PRADO for conducting the study and hence the assessment of the non-clinical safety of WSWPE was performed in a blinded manner at the test facility.

### Experimental animals and husbandry

Male and female Sprague–Dawley rats (aged 6–8 weeks), were sourced from National Institute of Bioscience, Pune, India (CPCSEA Registration Number: 1091/GO/Bt/S/07/CPCSEA). Rats allocated to one group were accommodated together in Individually-Ventilated-Cages (Optirat® Plus, Animal Care Systems, Inc., USA; Cage dimensions: 41 cm × 41 cm × 78 cm) with sterilized corn cob as the bedding material. The temperature in the experimental animal room was maintained between 20.6 and 22.7 °C while the relative humidity varied from 40 to 62%. The air changes in the animal room ranged from 10 to 15 per hour and the photoperiod was a 12-h light- dark cycle. Animals were offered pelleted laboratory animal diet (Nutrivet Lifescience, India) ad libitum, which was ultraviolet light sterilized. Additionally, they were supplied with ultraviolet light irradiated and reverse osmosis purified drinking water in steam-sterilized polypropylene bottles. After receiving the experimental animals from the supplier, rats were transferred to a quarantine room, where they were housed for a period of five days.

### Ethics declaration

The experimental procedures and animal husbandry practices strictly conformed to the CPCSEA standards^9^ and approval of the Institutional Animal Ethics Committee (IAEC) of the study site was in place (vide Protocol Number: IAEC-20–068), prior to the commencement of the study. Moreover, the study was conducted in compliance with The ARRIVE guidelines^10^.

### Experimental procedures

The non-clinical safety evaluation of WSWPE was conducted according to OECD test guideline number 407.

#### Animal acclimatization

After the completion of the quarantine period, 32 males and 32 females respectively, were allotted by the Animal Research Facility. Subsequently, the animals were shifted to an experimental room earmarked for the study, where they underwent acclimatization for five additional days. During the acclimatization phase, rats were differentiated by markings on the tail tips.

#### Animal randomization

Animals underwent a comprehensive clinical assessment after which, they were weighed by utilizing an animal weighing balance. Subsequently, 30 males and 30 female animals were randomly assigned to the study groups, on the basis of their body weights as mentioned in Supplementary Figure S1. All the study groups comprised of five male and five female rats. The animals were designated a permanent animal number and marked on the base of the tail. At the commencement of the experiment, the variation in the body weight of animals of either sex, did not surpass ± 20% of the average weight. Subsequent to randomization, the experimental animals were subjected to ophthalmoscopic examination by employing an ophthalmoscope, subsequent to inducing mydriasis with 1% Tropicamide solution.

#### Formulation preparation and oral administration

WSWPE was weighed and then it was dispensed to a mortar. It was then subjected to trituration with a pestle, following which a small amount of 0.5% methyl cellulose (Loba Chemie Private Limited, India) was added with constant blending. The remaining volume of the vehicle was subsequently added in dropwise fashion with continuous stirring to obtain a homogenous suspension. All the formulations were prepared fresh, throughout the compound administration phase.

Rats assigned to group G2 (low dose), G3 (mid dose), G4 (high dose) and G4R (high dose-recovery) were administered a dose of 100, 300, 1000 and 1000 mg/kg/day, respectively by oral route. Control animals from the main (G1) as well as the recovery arm (G1R) received 0.5% Methyl Cellulose. The dose volume was fixed at 10 mL/kg body weight.

#### Animal observations

Animals were monitored once a day for the development of any clinically abnormal signs, and twice a day for any manifestation of morbidity or the occurrence of mortality. After termination of the vehicle/extract administration phase, the observation duration was expanded for an additional 14 days, for animals allocated to the recovery arm (G1R and G4R respectively). The clinical signs were recorded for their onset, severity, duration and reversibility. Rats were assessed once a week for detailed clinical observations up to the termination of the experiment, in both the arms of the study. Examinations encompassed, changes in skin, fur, eyes, and mucous membranes, occurrence of secretions and excretions and basic observations of autonomic activity^8^.

#### Body weights

Animals were weighed on Day 1, 8, 15, 22 and 28 for the main study arm. In the recovery arm, rats were also weighed on days 35 and 42. Furthermore, the fasting body weights of the animals in both the main and recovery arms were also documented on the day of necropsy.

#### Food consumption

Food intake in different study groups were noted once every week. The per capita food consumption for one week was derived by employing the following formula:

$$Food\,consumption\,per\,week\,({\text{g}}) = \frac{{Food\,offered\,({\text{g}}) - Food\, leftover\,({\text{g}})}}{Number\,of\,animals}$$

#### Ophthalmoscopic examination

Rats allotted to the main study arm, that were administered with the vehicle (G1) and the high dose of WSWPE (G4) were examined for any extract-related ocular abnormalities in the fourth week of the study. Mydriasis was induced in the animals with 1% Tropicamide solution following which, both the eyes were observed by utilizing an ophthalmoscope This examination was not conducted in animals that received the low and mid-dose of the extract, as no WSWPE-related changes were observed in high-dose group animals.

#### Clinical pathology observations

On days 28 and 42 the animals allotted to different groups in the main and the recovery arm respectively were fasted overnight. Subsequently, on days 29 and 43, blood was withdrawn from the retro-orbital sinuses of the rats under transient isoflurane anaesthesia to evaluate the effect of WSWPE on hematology, coagulation and clinical chemistry parameters.

##### Hematology

Blood was dispensed in vials that contained ethylene diamine tetra acetic acid, dipotassium salt as the anticoagulant. After proper mixing, the blood was aspirated in a Sysmex XP-100 Auto Analyzer (Sysmex Corporation, Japan) and the following parameters were quantified: Red Blood Cell count (RBC), Hematocrit (HCT), Mean Corpuscular Volume (MCV), Hemoglobin (HGB), Mean Corpuscular Hemoglobin (MCH), Mean Corpuscular Hemoglobin Concentration (MCHC), Platelet Count (PLT) and White Blood Cell count (WBC). Differential leukocyte and Reticulocyte enumeration were performed manually on blood smears by staining them with Giemsa and New Methylene Blue stain respectively.

##### Coagulation

Whole blood was collected in Sodium Citrate-added tubes. Prothrombin Time (PT) and Activated Partial Thromboplastin Time (APTT) were assessed on Erba ECL-105 Coagulation Analyzer (Erba Mannheim, Germany).

##### Clinical chemistry

Blood was transferred to vials containing heparin for plasma separation and tubes devoid of any anticoagulant for serum separation, respectively. Plasma and sera were obtained by centrifugation (3000 revolutions per minute for 10 min). The sera were employed for electrolyte analysis viz. Sodium (Na^+^), Potassium (K^+^) and Chloride (Cl^−^), by utilizing a Sensacore Electrolyte Analyzer ST-200CL (Sensacore, India). The plasma samples were utilized for using for the estimation of the following parameters by employing an Erba EM Destiny 180 Auto Analyzer (Erba Mannheim, Germany): Glutamate Oxaloacetate Transaminase (GOT or AST), Glutamate Pyruvate Transaminase (GPT or ALT), Alkaline Phosphatase (ALP), Total Bilirubin (BILT), Blood Urea Level (BUL), calculated Blood Urea Nitrogen (BUN), Creatinine (CREAT), Glucose (GLU), Total Cholesterol (CHOLE), Total Protein (PRO), Albumin (ALB) and Protein: Albumin ratio (PAR).

##### Urinalysis

Qualitative urinalysis parameters were measured in overnight urine samples obtained from all the animals allocated to the various groups in both the main and recovery arms during the last week of treatment and recovery periods respectively, by employing metabolic cages for urine collection and Erba LAURA SMART urine analyzer (Erba Mannheim, Germany) for analysis. The parameters evaluated were: Bilirubin (BIL), glucose (GLU), protein (PRO), pH, and specific gravity (SG). Additionally, urine was manually examined for its colour and clarity.

#### Necropsy and macroscopic observation

Animals were humanely euthanized by asphyxiation induced by carbon dioxide. All the rats were subjected to a comprehensive macroscopic pathological examination, which incorporated an exhaustive scrutiny of the external surface of the body, all orifices, and the cranial, thoracic and abdominal cavities with their contents^8^.

#### Harvesting of organs and organ weighing

After gross pathology observations, the liver, kidneys, adrenals, testes/ovaries, thymus, spleen, brain and heart of all animals were dissected out and trimmed to remove any adherent tissue. Thereafter, their wet weights were noted and all the paired organs were weighed jointly. The organs selected for weighing were then transferred to 10% Neutral Buffered Formalin solution except the testes which were fixed in Modified Davidson’s Fixative Relative organ weight, expressed as a percentage of the fasting body weight, were then calculated for every individual animal by employing following formula:

$$Relative\,organ\,weight\,({\text{g}}) = \frac{{Organ\,weight\,({\text{g}})}}{{Terminal\,body\,weight\,({\text{g}})}} \times 100$$

Following organs were also harvested from all the animals and fixed in 10% Neutral Buffered Formalin for the ensuing microscopic examination: Aorta, Bone (Bone marrow), Cerebrum, Cerebellum, Midbrain, Epididymis, Esophagus, Large Intestine, Lungs, Lymph nodes (mesenteric), Pancreas, Sciatic nerve, Pituitary, Seminal Vesicle, Prostate, Skeletal Muscle, Skin, Small Intestine, Stomach, Spinal cord, Thyroid and Parathyroid, Trachea, Urinary bladder, Vagina, Uterus and Mammary glands. For the fixation of the eyes, Modified Davidson’s Fixative was used.

#### Histology

The fixed organs from the animals that received vehicle (G1) and high dose group (G4) of WSWPE from the main study arm underwent standard tissue processing procedures. Subsequently, by utilizing a tissue embedding station the tissues were embedded in paraffin. Thereafter, tissue sections of 3–5 µm thickness were obtained by employing a microtome and these sections were further stained with hematoxylin–eosin. The stained sections were thereafter evaluated for any observed microscopic alterations by utilizing microscope.

### Data analysis

Data was expressed as mean ± standard deviation from all the groups and were statistically analyzed by using Graph Pad Prism (Version 7.03). For the main study arm the body weights, hematological, coagulation, biochemistry, urinalysis (specific gravity and urinary pH) parameters and relative organ weights were analyzed using one-way ANOVA. When ANOVA was found to be statistically significant, Dunnett’s multiple comparison test was employed as the post-hoc test to ascertain the groups that were significantly different from the vehicle-administered groups at the 5% level i.e. *p* < 0.05. For the recovery arm, the aforementioned parameters were analyzed by employing Student’s *t* test for any significant difference at the 5% level between the group that received the vehicle (G1R) and the group that received the high-dose of WSWPE (G4R).

## Results

### Constituents and stability analysis of WSWPE suspension

**Figure 1:**
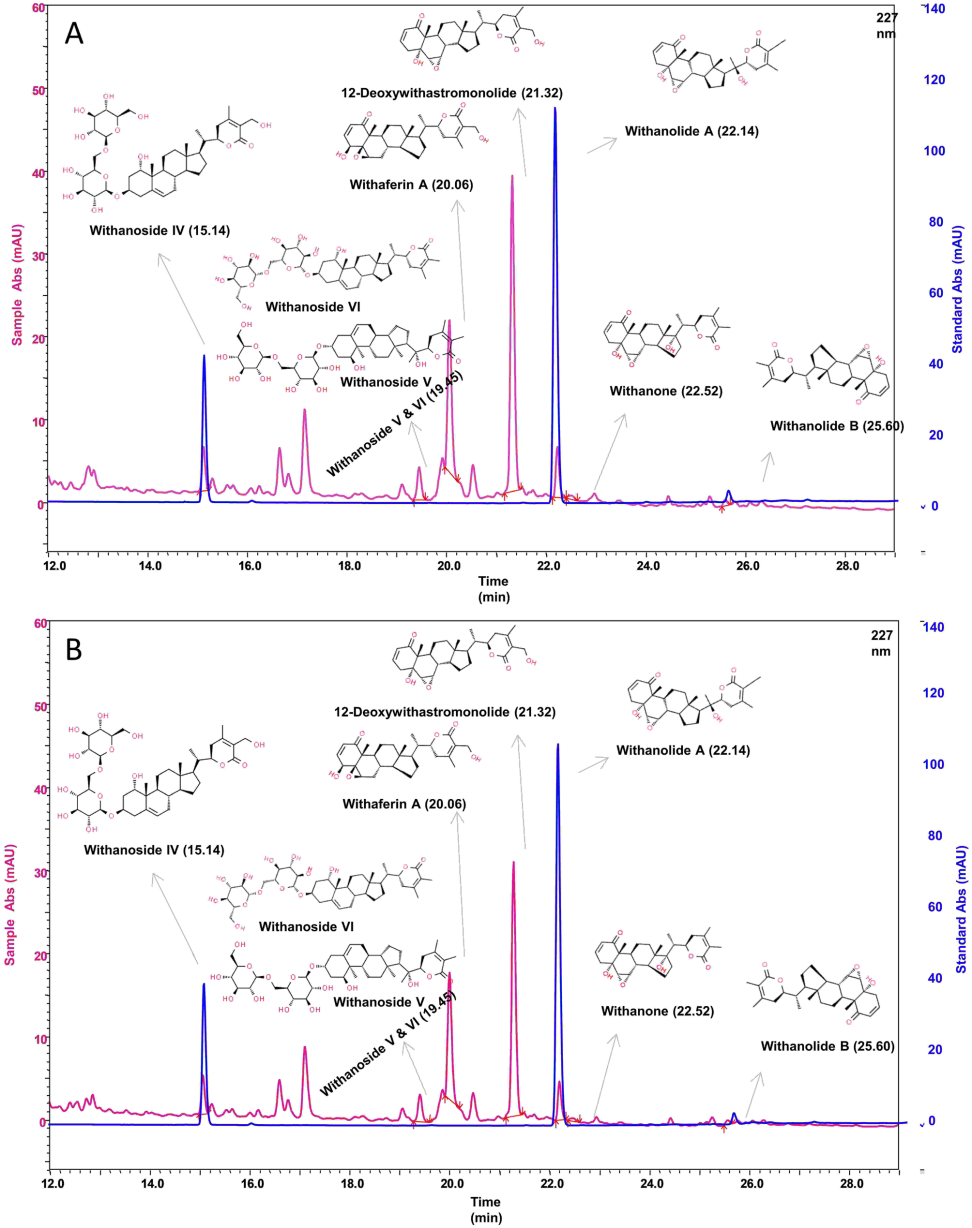
UHPLC chromatogram of phytoconstituents present in a suspension of WSWPE prepared by employing 0.5% methylcellulose, immediately (**A**) and after retaining it for 24 h at room temperature (**B**).

As illustrated in Fig. 1, the UHPLC chromatogram depicts that the formulated suspension of WSWPE, meant for oral administration to rats, comprised of Withanoside IV, Withaferin A, 12-Deoxywithastramonolide, Withanolide A, Withanone, Withanolide B, Withanoside V & VI. These phytoconstituents detected in the suspension are reported to be present in *Withania somnifera*^2^ which clearly characterizes the extract, chemically.

The concentrations of Withanoside IV at the initial stage and 24 h after maintaining the suspension at room temperature were 0.122 mg/mL and 0.106 mg/mL respectively (Supplementary Table S1). Further, the concentration of Withaferin A at both the aforementioned time points were 0.257 and 0.211 mg/mL, respectively (Supplementary Table S1). The concentration of the total Withanolides in the suspension were 0.885 mg/mL, immediately after preparation and 0.735 mg/mL after 24 h (Supplementary Table S1). Consequently, WSWPE was stable in the vehicle for at least 24 h at room temperature (Fig. 1, Supplementary Table S1). In the present study, however, we have particularly used freshly prepared suspensions for administering it to the rats on each day during the compound administration phase.

### Mortality and clinical signs

WSWPE administered by oral route did not lead to any mortality, morbidity, or abnormal clinical symptoms, in both male and female rats that were allocated to the 28-day treatment and the 14-day recovery groups (Supplementary Table S2). Further, the detailed clinical examinations, that were conducted every week, did not divulge any clinical abnormalities, which could be associated with the extract, in any animal throughout the study duration (Supplementary Table S3).

### Effects of WSWPE on body weights and food consumption

**Figure 2:**
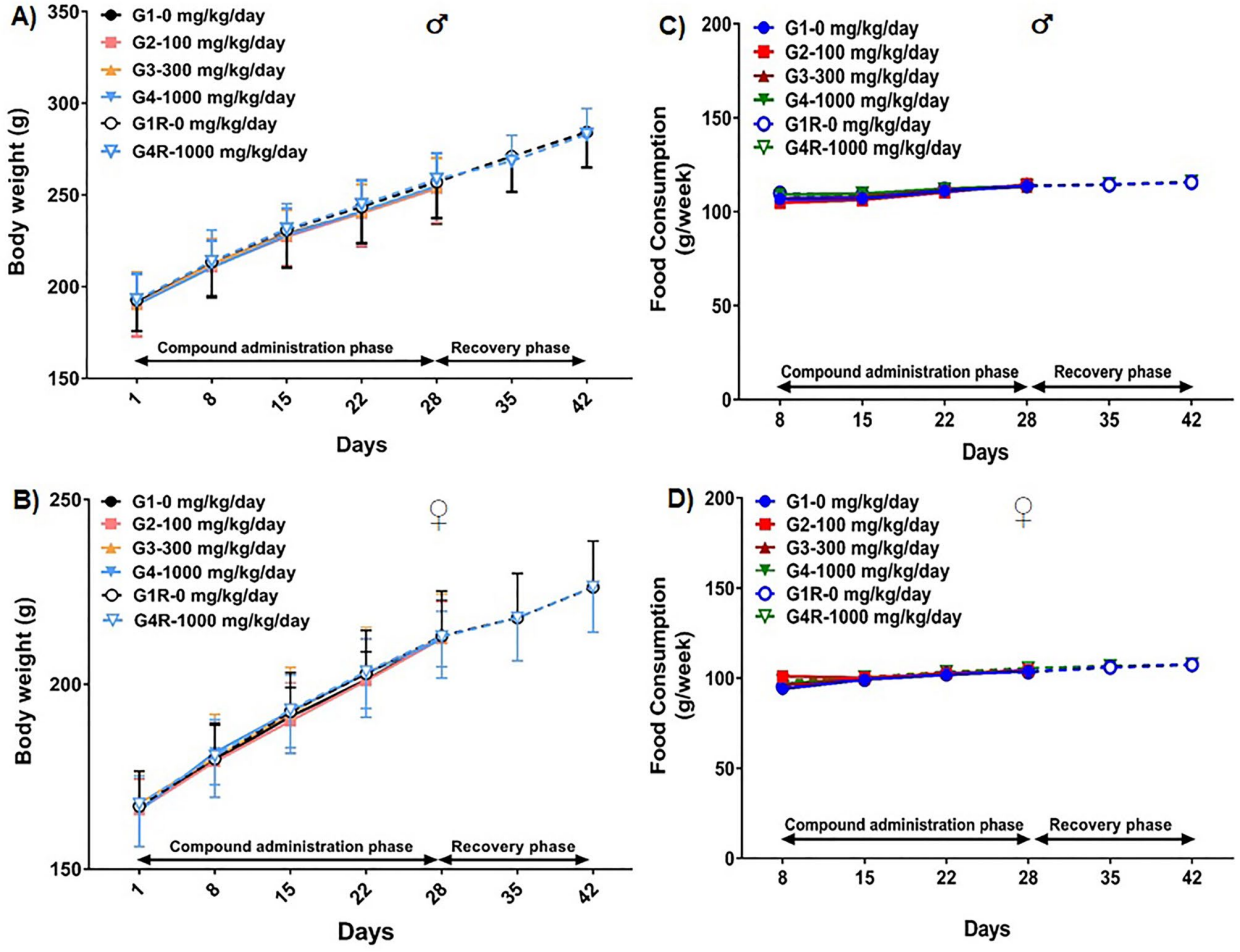
Body weight (Mean ± Standard Deviation) and food intake (Mean) of male (**A**, **C**, respectively) and female (**B**, **D**, respectively) rats, orally administered with WSWPE at the doses of 0, 100, 300 and 1000 mg/kg/day, for 28 days followed by a 14-day treatment free recovery period as elaborated in the methods.

When compared to their respective control groups, oral administration of WSWPE did not result in any statistically significant changes, in the absolute body weights of the animals of either sex, assigned to both the main and the recovery groups (Fig. 2A, B). Likewise, the weekly food consumption in the rats of all the treatment groups were comparable (Fig. 2C, D).

### Ophthalmic effects of WSWPE

Rats of either sex, that were assigned to the control and high dose groups, from the main study, were evaluated for any anomalous ocular outcomes in the last week of compound/vehicle administration. WSWPE did not ensue in any noticeable abnormality (Supplementary Table S4), when compared to the vehicle-administered group. Consequently, the animals that were administered with the low and mid-dose of the extract, were not examined.

### Effect of WSWPE on hematological parameters

The hematological parameters in the animals from both the 28-day treatment and the 14-day recovery groups, that received WSWPE were, for the most part, similar to their respective control groups (Table 1). However, amidst the rats allocated to the main study groups, a statistically significant increase in white blood cell count and mean corpuscular volume along with a significant decrease in platelet count was noted in male rats, which were administered the mid-dose of WSWPE (300 mg/kg/day), when compared to the control group (*p* < 0.05). These observations cannot be directly linked to WSWPE administration due to lack of dose-dependency and can be accordingly categorized as incidental findings. In the recovery arm, the male animals administered with WSWPE demonstrated a statistically significant decrease (*p* < 0.05) in total erythrocyte count and hematocrit when compared with the control group. These findings cannot be explicitly attributed to WSWPE as they are within the historical reference ranges for control animals^11^ and at the site of study. Moreover, these findings were not corroborated in female animals, allocated to the recovery arm.

**Table 1:** Effect of WSWPE on hematological and blood clotting parameters in rats.

Parameter	28 days treatment (mg/kg/day)				14 days recovery (mg/kg/day)	
	G1 (0)	G2 (100)	G3 (300)	G4 (1000)	G1R (0)	G4R (1000)
**Males**						
RBC (10^6^/µL)	7.60 ± 0.05	7.74 ± 0.12	6.50 ± 1.64	7.67 ± 0.24	8.18 ± 0.41	6.73 ± 1.15^#^
HGB (g/dL)	13.40 ± 0.00	13.42 ± 0.22	12.60 ± 2.64	13.64 ± 0.48	13.96 ± 0.64	11.90 ± 2.14
HCT (%)	41.38 ± 0.31	42.04 ± 0.28	38.30 ± 6.82	42.08 ± 1.87	43.48 ± 2.35	39.42 ± 2.18^#^
MCV (fL)	54.46 ± 0.09	54.34 ± 0.62	60.00 ± 5.41*	54.84 ± 0.71	53.14 ± 0.92	59.94 ± 11.08
MCH (pg)	17.64 ± 0.13	17.36 ± 0.53	19.62 ± 1.42	17.78 ± 0.13	17.08 ± 0.54	17.66 ± 0.55
MCHC (g/dL)	32.36 ± 0.26	31.92 ± 0.63	32.78 ± 1.39	32.42 ± 0.39	32.12 ± 0.82	30.20 ± 5.09
PLT (10^3^/µL)	747.00 ± 15.92	762.80 ± 151.91	534.60 ± 112.18*	696.60 ± 35.18	851.40 ± 212.34	833.20 ± 78.71
RET (%)	1.12 ± 0.23	1.08 ± 0.23	1.08 ± 0.30	1.00 ± 0.20	1.08 ± 0.23	1.04 ± 0.26
WBC (10^3^/µL)	13.38 ± 0.16	12.36 ± 0.21	17.52 ± 2.44*	13.00 ± 1.12	18.00 ± 3.57	16.50 ± 4.37
NEU (%)	25.80 ± 2.39	26.40 ± 2.51	26.00 ± 1.87	26.00 ± 1.58	27.20 ± 1.92	26.60 ± 2.07
LYM (%)	72.20 ± 2.28	71.80 ± 1.92	72.40 ± 2.07	72.20 ± 2.28	71.00 ± 1.58	71.60 ± 1.95
MONO (%)	1.20 ± 0.84	0.60 ± 0.55	0.60 ± 0.89	0.80 ± 0.84	0.60 ± 0.55	0.80 ± 0.84
EOS (%)	0.60 ± 0.89	1.00 ± 0.71	1.00 ± 0.71	0.80 ± 0.84	1.20 ± 0.84	0.80 ± 0.84
BASO (%)	0.20 ± 0.45	0.20 ± 0.45	0.00 ± 0.00	0.20 ± 0.45	0.00 ± 0.00	0.20 ± 0.45
PT (sec)	19.29 ± 2.95	16.15 ± 0.89	18.90 ± 1.43	14.50 ± 1.99*	16.26 ± 3.13	16.03 ± 1.85
APTT (sec)	20.90 ± 4.83	20.58 ± 3.25	18.76 ± 2.70	20.48 ± 1.11	19.99 ± 2.41	20.69 ± 1.80
**Females**						
RBC (10^6^/µL)	6.20 ± 0.95	6.70 ± 0.84	6.02 ± 0.02	5.91 ± 0.12	7.31 ± 0.58	7.97 ± 0.38
HGB (g/dL)	11.94 ± 1.15	12.80 ± 1.73	11.68 ± 0.04	11.22 ± 0.22	13.20 ± 1.31	13.98 ± 0.49
HCT (%)	38.02 ± 2.51	37.82 ± 5.50	45.12 ± 0.34*	41.52 ± 0.76	40.22 ± 2.30	42.56 ± 2.47
MCV (fL)	61.94 ± 5.35	56.38 ± 1.18*	75.00 ± 0.38*	70.24 ± 0.15*	55.44 ± 2.14	53.36 ± 0.75
MCH (pg)	19.42 ± 1.69	19.10 ± 0.31	19.42 ± 0.11	18.98 ± 0.25	18.16 ± 0.83	17.54 ± 0.34
MCHC (g/dL)	31.38 ± 1.31	33.90 ± 0.51*	25.90 ± 0.19*	27.04 ± 0.26*	32.76 ± 1.76	32.88 ± 0.86
PLT (10^3^/µL)	798.20 ± 159.93	850.80 ± 67.13	672.60 ± 3.58	550.00 ± 266.23	993.60 ± 203.13	888.80 ± 107.36
RET (%)	1.04 ± 0.17	1.04 ± 0.26	1.08 ± 0.23	1.00 ± 0.14	1.08 ± 0.23	1.04 ± 0.17
WBC (10^3^/µL)	12.04 ± 1.88	13.40 ± 5.39	13.34 ± 0.11	15.20 ± 0.64	14.74 ± 3.95	15.32 ± 2.66
NEU (%)	25.80 ± 2.39	26.40 ± 2.51	26.00 ± 1.87	26.00 ± 1.58	27.20 ± 1.92	26.60 ± 2.07
LYM (%)	72.20 ± 2.28	71.80 ± 1.92	72.40 ± 2.07	72.20 ± 2.28	71.00 ± 1.58	71.60 ± 1.95
MONO (%)	1.20 ± 0.84	0.60 ± 0.55	0.60 ± 0.89	0.80 ± 0.84	0.60 ± 0.55	0.80 ± 0.84
EOS (%)	0.60 ± 0.89	1.00 ± 0.71	1.00 ± 0.71	0.80 ± 0.84	1.20 ± 0.84	0.80 ± 0.84
BASO (%)	0.20 ± 0.45	0.20 ± 0.45	0.00 ± 0.00	0.20 ± 0.45	0.00 ± 0.00	0.20 ± 0.45
PT (sec)	17.79 ± 3.21	18.95 ± 1.05	18.02 ± 0.48	14.07 ± 1.48*	16.75 ± 1.37	18.36 ± 2.31
APTT (sec)	16.91 ± 2.71	18.29 ± 0.34	18.50 ± 1.60	18.53 ± 1.29	18.61 ± 1.95	21.18 ± 1.16^#^

Data is presented as Mean ± Standard Deviation (n = 5 animals per group per sex). *p < 0.05 vs. G1 (One-Way ANOVA followed by Dunnett’s post-hoc multiple comparison test), ^#^*p* < 0.05 vs. G1R (Student’s *t* test).

RBC, red blood cell count; HGB, Hemoglobin; HCT, hematocrit; MCV, mean corpuscular volume; MCH, mean corpuscular hemoglobin; MCHC, mean corpuscular hemoglobin concentration; PLT, platelet; RET, reticulocyte; WBC, white blood cell count; NEU, neutrophils, LYM, lymphocytes; MONO, monocytes; EOS, eosinophils; BASO, basophils; PT, prothrombin time; APTT, activated partial thromboplastin time.

In animals of the female gender that were allocated to the main study, the hematocrit was significantly elevated (*p* < 0.05) in rats that were administered with the mid-dose of WSWPE (300 mg/kg/day), when compared to the vehicle-administered group. Furthermore, as compared to the control group, the mean corpuscular volume was significantly decreased (*p* < 0.05) in the animals that received the low dose (100 mg/kg/day) but significantly higher (*p* < 0.05) in the rats that received the mid (300 mg/kg/day) and the high dose (1000 mg/kg/day) of WSWPE, respectively. Additionally, the mean corpuscular hemoglobin concentration was significantly increased (*p* < 0.05) in animals that were administered the low dose (100 mg/kg/day) but were significantly decreased in animals which received the mid (300 mg/kg/day) and high (1000 mg/kg/day) of the extract. All these observed alterations in female cannot be ascribed to WSWPE as they were not dose-dependent. Furthermore, they are unlikely to be of any clinical significance as the values are within the historical reference ranges for the control animals and at the study site.

Finally, no gross or histopathological changes were observed in the spleen or the bone marrow of animals that received the high dose of WSWPE, which could elucidate these hematological outcomes in both male and female rats.

### Effects of WSWPE on coagulation parameters

This study also examined the effect of WSWPE on blood clotting parameters including prothrombin time and activated partial thromboplastin time. Both the studied parameters were largely unaffected following the oral administration of WSWPE in both male and female in both the main and recovery arms (Table 1). Nevertheless, as compared to the control group, a minor, but statistically significant decrease (*p* < 0.05) in prothrombin time was observed in both male and female rats assigned to the main study arm, which were administered the high dose of WSWPE (1000 mg/kg/day). In female animals allocated to the recovery arm, a significant increase (*p* < 0.05) in activated partial thromboplastin time was noted in the WSWPE-administered animals. These, findings are improbable to be of any clinical significance as the values are well within the reference ranges described for SD rats and at the site of the study.

### Effect of WSWPE on clinical chemistry parameters

The clinical chemistry parameters were by and large unaltered subsequent to the oral administration of WSWPE in both male and female animals (Table 2). Nonetheless, in male animals assigned to the 28-day treatment groups, a significant increase (*p* < 0.05) in cholesterol levels were observed in rats that received the low (100 mg/kg/day) and high dose (1000 mg/kg/day) of WSWPE respectively, when compared to the control groups. Additionally, as compared to the control group, significantly elevated serum levels of Na^+^ were detected in rats that were administered the mid-dose (300 mg/kg/day) of the extract (*p* < 0.05). These observed changes were however not dose-related. Further, WSWPE administration to male rats at the dose of 1000 mg/kg/day, lead to a significant increase in total bilirubin level (*p* < 0.05), when compared to the control group. This effect, although dose-related, is unlikely to be of any clinical relevance as the recorded level was well within the reference range described for SD rats and at the study site. Similarly, in the recovery arm a significant increase in alkaline phosphatase value (*p* < 0.05) was detected in WSWPE-administered animals, as compared to the control group and this level was also within the historical reference ranges, thereby eliminating the risk for a potential translation to any clinical consequence.

**Table 2:** Effect of WSWPE on clinical chemistry parameters in rats.

Parameter	28 days treatment (mg/kg/day)				14 days recovery (mg/kg/day)	
	G1 (0)	G2 (100)	G3 (300)	G4 (1000)	G1R (0)	G4R (1000)
**Males**						
GPT (U/L)	84.90 ± 10.27	83.97 ± 10.85	81.50 ± 22.56	73.23 ± 12.32	55.09 ± 11.81	45.90 ± 12.02
GOT (U/L)	124.24 ± 23.08	108.27 ± 22.59	97.43 ± 10.77	124.24 ± 23.08	108.27 ± 22.59	97.43 ± 10.77
BUL (mg/dL)	34.42 ± 7.75	37.48 ± 5.44	38.05 ± 8.93	36.44 ± 7.04	33.32 ± 3.99	29.75 ± 4.00
BUN (mg/dL)	16.08 ± 3.62	17.51 ± 2.54	17.78 ± 4.17	17.03 ± 3.29	15.57 ± 1.86	13.90 ± 1.87
CREAT (mg/dL)	0.83 ± 0.29	0.57 ± 0.14	0.57 ± 0.18	0.59 ± 0.11	0.86 ± 0.07	0.82 ± 0.05
GLU (mg/dL)	118.48 ± 28.02	133.10 ± 27.56	107.94 ± 9.65	116.54 ± 20.68	93.59 ± 12.04	93.26 ± 13.87
CHOLE (mg/dL)	61.50 ± 9.26	77.44 ± 8.93*	67.53 ± 3.43	75.98 ± 10.19*	65.05 ± 12.42	69.86 ± 23.34
PRO (g/dL)	7.22 ± 0.44	7.57 ± 0.41	6.46 ± 0.55	7.94 ± 0.70	5.81 ± 1.11	6.26 ± 0.30
ALB (g/dL)	2.62 ± 0.13	2.57 ± 0.26	2.31 ± 0.21	2.65 ± 0.27	2.26 ± 0.25	2.15 ± 0.27
PAR	2.76 ± 0.29	2.97 ± 0.40	2.82 ± 0.38	3.04 ± 0.49	2.59 ± 0.60	2.94 ± 0.32
ALP (U/L)	203.50 ± 52.27	144.32 ± 52.38	180.38 ± 54.86	197.95 ± 65.11	118.60 ± 12.16	157.00 ± 33.82^#^
BILT (mg/dL)	0.21 ± 0.08	0.27 ± 0.07	0.30 ± 0.03	0.35 ± 0.03*	0.16 ± 0.01	0.28 ± 0.15
Na^+^ (mmol/L)	140.92 ± 1.01	140.52 ± 1.09	144.62 ± 2.17*	140.80 ± 1.22	136.36 ± 1.49	134.66 ± 0.96
K^+^ (mmol/L)	7.64 ± 0.97	7.40 ± 0.95	6.59 ± 0.97	7.94 ± 1.46	12.90 ± 1.10	12.13 ± 0.76
Cl^−^ (mmol/L)	100.76 ± 1.11	100.26 ± 0.87	103.24 ± 1.85	100.76 ± 2.08	100.82 ± 4.25	97.16 ± 1.72
**Females**						
GPT (U/L)	84.15 ± 20.44	70.12 ± 6.39	80.76 ± 6.84	91.48 ± 25.73	52.26 ± 7.53	43.28 ± 4.97
GOT (U/L)	149.50 ± 20.35	122.12 ± 16.05	152.88 ± 31.40	127.37 ± 24.10	100.97 ± 7.01	111.65 ± 18.31
BUL (mg/dL)	36.44 ± 3.57	29.35 ± 5.51	34.71 ± 6.58	30.04 ± 1.45	30.79 ± 4.26	28.71 ± 3.94
BUN (mg/dL)	17.03 ± 1.67	13.71 ± 2.57	16.22 ± 3.07	14.04 ± 0.68	14.39 ± 1.99	13.41 ± 1.84
CREAT (mg/dL)	0.67 ± 0.10	0.73 ± 0.13	0.63 ± 0.07	0.65 ± 0.14	0.75 ± 0.06	0.81 ± 0.08
GLU (mg/dL)	97.65 ± 13.94	102.67 ± 35.28	127.22 ± 37.61	106.87 ± 29.64	88.80 ± 20.97	89.45 ± 15.14
CHOLE (mg/dL)	67.04 ± 7.36	66.09 ± 19.24	70.82 ± 16.48	77.28 ± 11.88	72.32 ± 15.69	63.91 ± 14.43
PRO (g/dL)	7.31 ± 0.61	7.70 ± 0.60	7.21 ± 1.05	7.58 ± 0.35	5.47 ± 0.65	6.00 ± 0.52
ALB (g/dL)	2.77 ± 0.33	2.72 ± 0.28	2.84 ± 0.22	2.74 ± 0.15	2.34 ± 0.24	2.24 ± 0.09
PAR	2.67 ± 0.40	2.86 ± 0.42	2.55 ± 0.43	2.78 ± 0.27	2.36 ± 0.45	2.68 ± 0.30
ALP (U/L)	147.42 ± 42.82	178.02 ± 46.62	204.08 ± 41.26	157.76 ± 36.77	147.40 ± 51.00	149.20 ± 35.09
BILT (mg/dL)	0.23 ± 0.08	0.30 ± 0.03	0.36 ± 0.02*	0.23 ± 0.07	0.16 ± 0.07	0.27 ± 0.11
Na^+^ (mmol/L)	140.04 ± 1.17	140.72 ± 1.92	139.80 ± 2.35	139.86 ± 1.54	137.18 ± 0.89	134.92 ± 1.43^#^
K^+^ (mmol/L)	7.46 ± 1.00	6.95 ± 0.95	8.25 ± 2.79	8.55 ± 1.91	12.66 ± 1.37	12.74 ± 0.54
Cl^−^ (mmol/L)	102.78 ± 1.06	102.34 ± 0.71	102.84 ± 2.32	102.78 ± 0.58	100.78 ± 1.46	100.34 ± 3.08

Data is presented as Mean ± Standard Deviation (n = 5 animals per group per sex). *p < 0.05 vs. G1 (One-Way ANOVA followed by Dunnett’s post-hoc multiple comparison test), ^#^*p* < 0.05 vs. G1R (Student’s *t* test).

GPT, glutamate pyruvate transaminase; GOT, glutamate oxaloacetate transaminase; BUL, blood urea level; BUN, blood urea nitrogen; CREAT, creatinine; GLU, glucose; CHOLE, total cholesterol; PRO, total protein; ALB, albumin; PAR, protein albumin ration; BILT, total bilirubin.

In female animals that were allocated to the main study arm, a significant elevation in total bilirubin level was observed in rats (*p* < 0.05) that received the mid-dose (300 mg/kg/day) of WSWPE. This finding however was not dose-dependent. In female rats assigned to the recovery arm, a statistically significant decrease (*p* < 0.05) in the serum Na^+^ level was observed, when compared to the corresponding control group. Nevertheless, this change was well within the reference range and at the site of the study.

### Effect of WSWPE on qualitative urinalysis parameters

Oral administration of WSWPE in rats of either sex, assigned to both the main and recovery study arms, did not have a significant effect on the urinalysis parameters when compared to their respective control groups (Table 3).

**Table 3:** Effect of WSWPE on qualitative urinalysis parameters in rats.

Parameter	28 days treatment (mg/kg/day)				14 days recovery (mg/kg/day)	
	G1 (0)	G2 (100)	G3 (300)	G4 (1000)	G1R (0)	G4R (1000)
**Males**						
Specific gravity	1.025 ± 0.011	1.030 ± 0.000	1.030 ± 0.000	1.024 ± 0.013	1.003 ± 0.003	1.003 ± 0.003
pH	6.20 ± 0.67	5.60 ± 0.82	5.60 ± 0.82	5.60 ± 0.82	6.70 ± 0.27	6.70 ± 0.27
Colour: yellow	5/5	5/5	5/5	5/5	5/5	5/5
Turbidity: clear	5/5	5/5	5/5	5/5	5/5	5/5
**Bilirubin**						
(a) Negative	5/5	5/5	5/5	5/5	5/5	5/5
(b) Positive	0/5	0/5	0/5	0/5	0/5	0/5
**Protein**						
(a) Negative	5/5	5/5	5/5	5/5	5/5	5/5
(b) Positive	0/5	0/5	0/5	0/5	0/5	0/5
**Glucose**						
(a) Negative	5/5	5/5	5/5	5/5	5/5	5/5
(b) Positive	0/5	0/5	0/5	0/5	0/5	0/5
**Females**						
Specific gravity	1.024 ± 0.013	1.024 ± 0.013	1.030 ± 0.000	1.030 ± 0.000	1.000 ± 0.000	1.000 ± 0.000
pH	6.00 ± 0.61	6.10 ± 0.65	6.50 ± 0.00	6.20 ± 0.67	6.60 ± 0.22	6.50 ± 0.00
Colour: yellow	5/5	5/5	5/5	5/5	5/5	5/5
Turbidity: clear	5/5	5/5	5/5	5/5	5/5	5/5
**Bilirubin**						
(a) Negative	5/5	5/5	5/5	5/5	5/5	5/5
(b) Positive	0/5	0/5	0/5	0/5	0/5	0/5
**Protein**						
(a) Negative	5/5	5/5	5/5	5/5	5/5	5/5
(b) Positive	0/5	0/5	0/5	0/5	0/5	0/5
**Glucose**						
(a) Negative	5/5	5/5	5/5	5/5	5/5	5/5
(b) Positive	0/5	0/5	0/5	0/5	0/5	0/5

Data is presented as Mean ± Standard Deviation for specific gravity and pH and number of animals for yellow colour, no turbidity and for presence or absence of bilirubin, protein and glucose in the urine.

### Relative organ weights in rats administered with WSWPE

The relative organ weights of WSWPE-administered male and female rats, allocated to both the main as well as the recovery groups, were comparable to those of their respective control groups (Table 4).

**Table 4:** Effect of WSWPE on relative organ weights expressed as a percentage of the fasting body weights.

Parameter	28 days treatment (mg/kg/day)				14 days recovery (mg/kg/day)	
	G1 (0)	G2 (100)	G3 (300)	G4 (1000)	G1R (0)	G4R (1000)
**Males**						
Body weight (g)	243.00 ± 19.36	241.90 ± 18.97	243.20 ± 15.69	242.60 ± 18.35	274.40 ± 18.28	274.10 ± 13.18
Liver	3.641 ± 0.327	3.521 ± 0.107	3.467 ± 0.064	3.649 ± 0.256	3.272 ± 0.274	3.345 ± 0.624
Spleen	0.349 ± 0.029	0.334 ± 0.024	0.338 ± 0.026	0.334 ± 0.032	0.283 ± 0.069	0.290 ± 0.078
Heart	0.332 ± 0.019	0.331 ± 0.010	0.334 ± 0.007	0.335 ± 0.004	0.295 ± 0.016	0.308 ± 0.056
Thymus	0.082 ± 0.006	0.083 ± 0.005	0.083 ± 0.002	0.085 ± 0.004	0.062 ± 0.010	0.066 ± 0.024
Kidneys	0.860 ± 0.052	0.858 ± 0.068	0.855 ± 0.032	0.858 ± 0.058	0.770 ± 0.083	0.848 ± 0.183
Adrenals	0.015 ± 0.001	0.014 ± 0.001	0.016 ± 0.001	0.015 ± 0.001	0.011 ± 0.002	0.012 ± 0.003
Testes	1.165 ± 0.164	1.108 ± 0.109	1.114 ± 0.074	1.160 ± 0.071	1.044 ± 0.092	1.010 ± 0.084
Brain	0.777 ± 0.045	0.777 ± 0.052	0.760 ± 0.020	0.777 ± 0.058	0.649 ± 0.047	0.654 ± 0.090
**Females**						
Body weight (g)	202.40 ± 8.96	202.20 ± 8.70	202.00 ± 9.90	201.40 ± 7.44	216.60 ± 13.74	216.80 ± 11.42
Liver	3.258 ± 0.151	3.328 ± 0.376	3.294 ± 0.138	3.338 ± 0.398	2.819 ± 0.129	2.771 ± 0.272
Spleen	0.439 ± 0.066	0.408 ± 0.014	0.424 ± 0.017	0.426 ± 0.013	0.196 ± 0.038	0.227 ± 0.068
Heart	0.319 ± 0.019	0.336 ± 0.022	0.313 ± 0.011	0.339 ± 0.036	0.293 ± 0.011	0.298 ± 0.035
Thymus	0.102 ± 0.006	0.106 ± 0.008	0.108 ± 0.009	0.101 ± 0.007	0.073 ± 0.014	0.082 ± 0.020
Kidneys	0.874 ± 0.030	0.870 ± 0.017	0.874 ± 0.040	0.879 ± 0.015	0.659 ± 0.013	0.666 ± 0.021
Adrenals	0.029 ± 0.002	0.029 ± 0.002	0.029 ± 0.002	0.029 ± 0.001	0.023 ± 0.001	0.021 ± 0.006
Testes	0.044 ± 0.003	0.044 ± 0.003	0.043 ± 0.001	0.043 ± 0.003	0.028 ± 0.003	0.030 ± 0.004
Brain	0.878 ± 0.023	0.866 ± 0.042	0.864 ± 0.021	0.893 ± 0.031	0.783 ± 0.014	0.804 ± 0.065

Data is presented as Mean ± Standard Deviation. Body weight of animals presented in the table represents their fasting body weights on the day of necropsy.

### Gross pathological findings

Macroscopic examination of the organs harvested from male and female rats that received WSWPE, did not reveal any lesions of pathological relevance, both in the 28-day treatment arm as well as the 14-day recovery arm groups (Supplementary Table S5).

### Histological analysis

**Figure 3:**
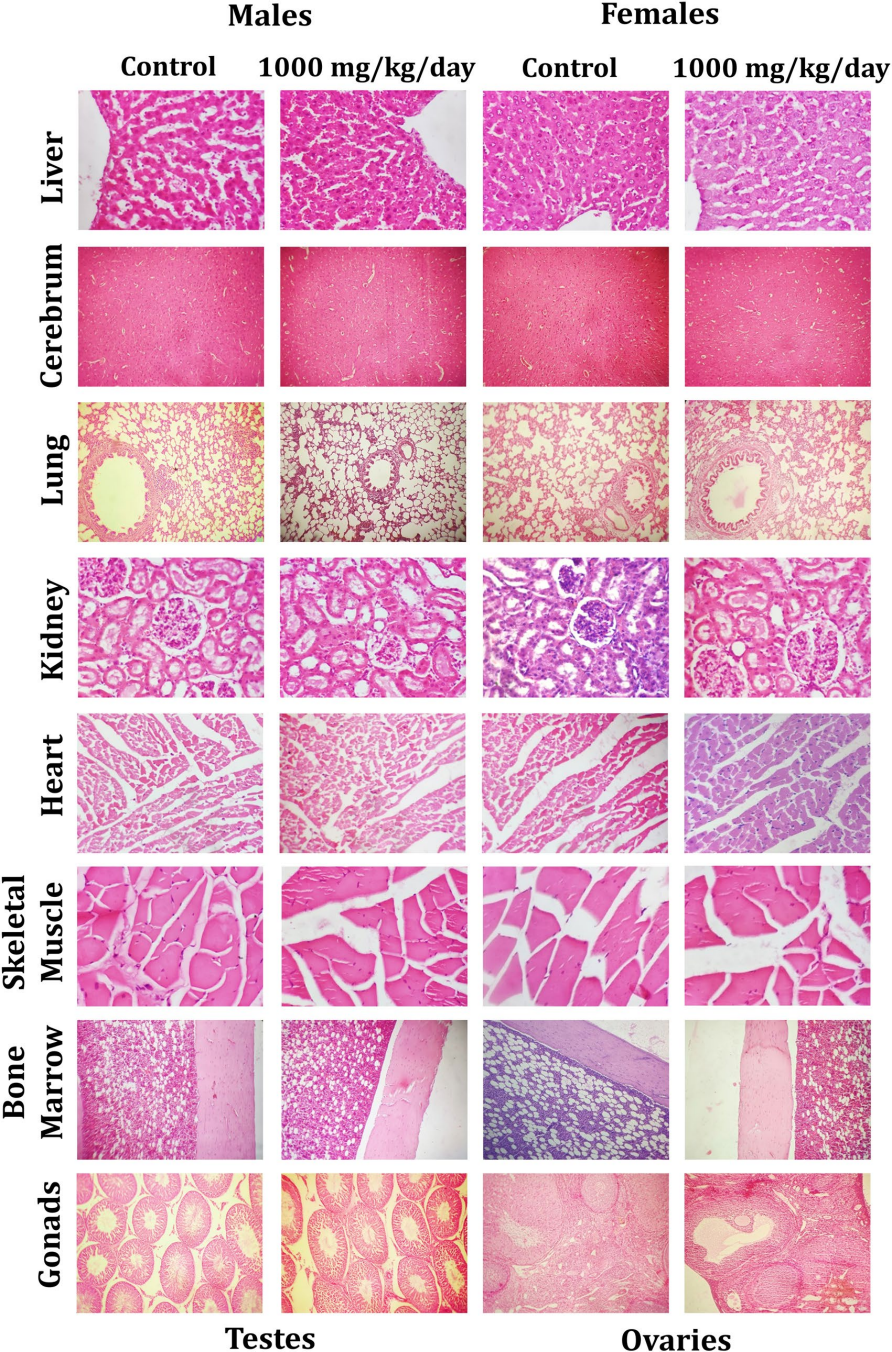
Representative histological photomicrographs of selected organs of rats that orally received either the vehicle (0.5% methylcellulose) or the high dose (1000 mg/kg/day) of WSWPE for a total duration of 28 consecutive days, in this non-clinical safety study. Tissue sections have been stained with Haematoxylin and Eosin. Following organs have been processed for imaging: Cerebrum, Lung, Bone Marrow (× 100); Liver, Kidney, Heart, Skeletal Muscle, Testis, Ovary (× 400).

**Figure 4:**
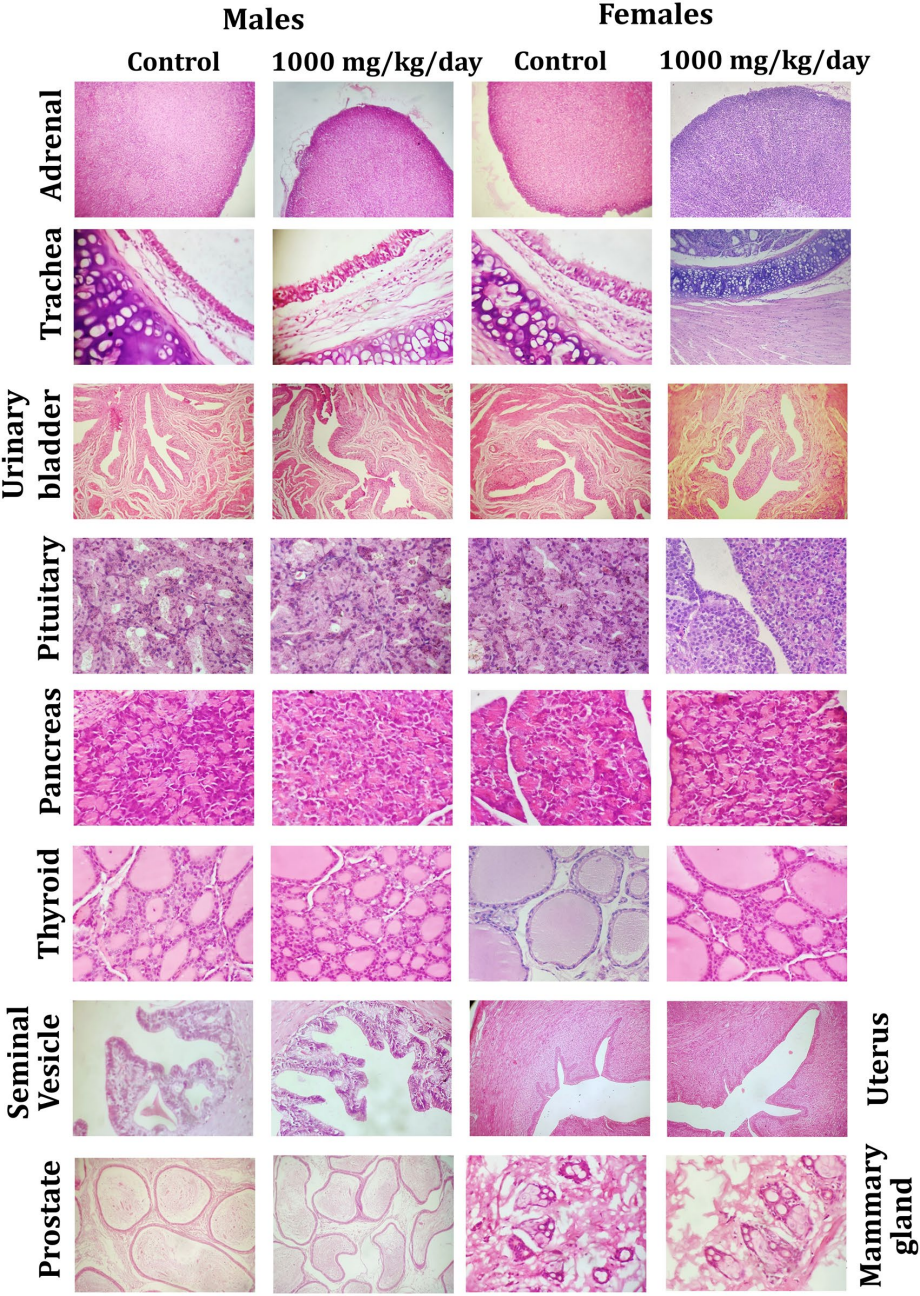
Representative histological photomicrographs of selected organs of rats that orally received either the vehicle (0.5% methylcellulose) or the high dose (1000 mg/kg/day) of WSWPE for a total duration of 28 consecutive days, in this non-clinical safety study. Tissue sections have been stained with Haematoxylin and Eosin. Adrenal gland (× 40); Trachea, Urinary Bladder, Uterus (× 100); Pituitary, Pancreas, Thyroid, Seminal Vesicle, Prostate, Mammary Gland (× 400).

Microscopic evaluation of tissues was performed in male and female rats of the main study group, which were administered the vehicle and the high dose of WSWPE (1000 mg/kg/day) respectively. The animals which received the high dose of WSWPE, did not exhibit any abnormal histological characteristics that can be specifically ascribed to the extract (Fig. 3 and Fig. 4; Supplementary Table S6).

## Discussion

Although the roots of *Withania somnifera* plant are primarily used for the preparation of classical Ayurvedic formulations, modern scientific investigations suggest that the aerial parts of the plant namely its leaves, stem, fruit and seeds also possess several biologically active phytoconstituents. The preclinical in-vitro and in-vivo pharmacological activities of the said aerial parts been studied with positive outcomes. To elaborate further, the leaf extracts of *Withania somnifera* plant have preclinically demonstrated several pharmacological activities like, anti-cancer^12–16^, anti-neuroinflammatory^17,18^, anxiolytic and sleep inducing^18,19^, cardioprotective^20^, anti-diabetic^21,22^, anti-microbial^23,24^, and anti-osteoporotic^25^. Moreover, in certain studies, the extracts prepared from the leaves have found to possess a superior activity when compared to the roots of *Withania somnifera*. To exemplify, a 50% ethanolic extract prepared from the leaves and stems of *Withania somnifera* have been reported to possess a superior in-vitro cytotoxic activity against several human cancer cell lines when compared to a similar extract prepared from the roots^26^.

Clinical investigations have also been conducted to assess the efficacy and safety of *Withania somnifera* formulations including the leaves. One such formulation consisting of the combined extracts of the leaves and roots has exhibited clinical efficacy in improving cognitive and psychomotor performance^27^ and reducing pain, joint stiffness and disability in osteoarthritis-afflicted patients^28^. The specific extracts of the berries of *Withania somnifera* are known to possess anti-oxidant^6^, and anti-cancer activities^29^. The *Withania somnifera* stem extracts are also known to possess anti-cancer activity^26^.

A recent study has further demonstrated that the fatty acids extracted from the seeds of *Withania somnifera* repaired 12-O tetradecanoyl phorbol 13-acetate (TPA)-induced psoriasis-like skin lesions in mice by modulating the release of pro-inflammatory cytokines, under in-vitro conditions^7^. Taken together, besides the roots, the other aerial parts of *Withania somnifera* plant also have therapeutic as well as prophylactic potentials from the clinical perspectives.

Consequently, with an objective to support the forthcoming non-clinical safety investigations in non-rodents and the future efficacy and safety trials in human subjects, we have assessed the safety profile of a hydromethanolic extract prepared from the whole plant of *Withania somnifera* (WSWPE) which incorporates the roots as well. We report here for the first time a GLP-driven subacute toxicity assessment, in which WSWPE was continually administered to male and female rats by oral route for a study period of 28 days followed by a 14-day extract administration free, recovery period, in a satellite group of animals.

In the present study, subsequent to WSWPE administration in rats of either sex, we did not observe any mortality or abnormal clinical signs; no significant effect on body weights and food intake and no ophthalmic aberrations as compared to the vehicle administered animals, in both the main and recovery arms. Similarly, at study termination, no gross pathological changes were observed in any organ and the organ weights were found to be comparable to the animals that received the vehicle alone. Microscopic examination of the organs collected from animals that were administered with the highest dose of the extract also did not reveal any WSWPE-related alterations. Likewise, the qualitative urinalysis parameters were also equivalent between the vehicle and WSWPE-administered groups in both the main and recovery arms. Nevertheless, we did detect some statistically significant changes in hematology, coagulation and clinical chemistry parameters. These alterations, in our view, were not considered to be adverse and moreover not explicitly related to WSWPE as they were neither non-dose dependent, nor explicable due to the non-existence or any macro- or microscopic findings in organs harvested at the time of necropsy. Furthermore, the findings are unlikely to be of any clinical significance, as the evaluated parameters in which statistical significance was noted were within the historical reference ranges described for SD rats at the site of the study. Consequently, as concluded from the study the NOAEL of WSWPE in SD rats was determined to be 1000 mg/kg/day.

Although we reiterate that the present study is the first description of the non-clinical safety evaluation of WSWPE, it is noteworthy to briefly describe the existing non-clinical safety profiles, available in the public domain for the extracts derived from the roots of *Withania somnifera* as well as its phytoconstituents.

The acute and subacute safety assessment of an ethanolic extract of *Withania somnifera* roots has been reported, wherein the extract was administered to mice and rats of either sex, respectively by intraperitoneal route^30^. In the acute toxicity study, the LD_50_ of the root extract was determined to be 1260 mg/kg, i.p. in mice. In the ensuing subacute toxicity assessment, male and female Wistar rats received the extract at the dose of 100 mg/kg, i.p. repeatedly for 30 days. No mortality was observed in any animal and as compared to the vehicle-control group, no significant differences were reported in food and water intake as well as in the body weight gain, subsequent to administration of the extract. However, significant decrease in absolute weights of spleen, adrenals and thymus were reported in only male animals. Examination of hematological parameters revealed a significant increase in hemoglobin concentration whereas the clinical chemistry parameters were largely unchanged. Finally, no gross-or histologic changes were detected in any organ in all the animals. In another acute toxicity study conducted in accordance with the OECD guideline 420, aqueous, hydroalcoholic and ethanolic extracts of the dried roots of *Withania somnifera* were administered orally to female albino mice, wherein all extracts were reported to be safe up to 2000 mg/kg, p.o.^31^. In another study an aqueous extract of the plant roots was administered to male Wistar rats orally at the dose of 3000 mg/kg for seven days, in which no extract-related deaths, apparent clinical signs of toxicity, stress, and adverse impact on food and water intake was detected^32^ The acute toxicity study evaluation of a root extract of *Withania somnifera*, standardized for Withaferin-A was orally administered to female Wistar rats by oral route at the doses of 500, 1000 and 2000 mg/kg^33^. In this study the LD_50_ was reported to be more than 2000 mg/kg and the extract was devoid of any behavioral effects and moreover no gross organ pathologies were revealed. The same study has furthermore examined the subacute toxicity of the same extract, in male and female rats that received the extract at doses of 500, 1000 and 2000 mg/kg. In this experiment as well no major toxicities were unearthed and the alterations observed in certain hematology and clinical chemistry parameters were within reference ranges defined for rats, as also observed in our study. Moreover, and no macro or microscopic organ pathologies were revealed and consequently, the NOAEL was regarded to be 2000 mg/kg. In one other related study, a hydroalcoholic extract prepared from the roots of *Withania somnifera* was evaluated for its acute as well as subacute toxicity^34^ and the study conclusions were precisely analogous to the previously quoted study^33^. Additionally, one study has assessed the sub-chronic toxicity of a pure *Withania somnifera* extract, wherein, the extract was orally administered to rats for a study duration of 90 days at the doses of 100, 500 and 1000 mg/kg/day^35^. In this study the NOAEL was adjudged to be 1000 mg/kg/day as the extract was devoid of any apparent toxicities up to this dose. Finally, an aqueous extract of the roots was determined to be non-toxic in a chronic toxicity study conducted in rats that were administered 250 mg/kg of the extract by oral route for 8 months^1^. Nevertheless, some findings of this study were significant increase in body weight and liver weight and a significant decrease in plasma cortisol levels.

Acute toxicity assessments of the bioactive components of *Withania somnifera*, which have also been detected in WSWPE, have also been conducted. The LD_50_ of Withaferin A in rats has been reported to be 670 mg/kg, by oral route and the LD_50_ of Withanone was computed to be more than 400 mg/kg in rats that received the pure phytoconstituent by oral route^1^. The high LD_50_ values reported for Withaferin A and Withanone further substantiate the acceptable safety profile observed in our study as the amount of these two constituents present in WSWPE are only a fraction of the reported LD_50_ values.

When compared to the published non-clinical safety profile of the extract of roots, in our study we did not observe any toxicities as well with the hydromethanolic extract obtained from the whole plant of *Withania somnifera*. We speculate that due to the similarity in the phytoconstituents of the roots and the whole plant, which include its aerial parts namely the leaves, stem, berries and seeds, a safety profile of the aerial parts comparable to the roots can be expected.

This observed acceptable subacute toxicity profile of WSWPE enables further assessment of its non-clinical safety in non-rodents and its sub-chronic and chronic toxicity assessment in rodents. Ultimately, based on the study outcomes, the efficacy and safety of WSWPE can also be extended to patients afflicted with disorders for which WSWPE is of potential therapeutic value.

## Conclusion

The non-clinical subacute toxicity evaluation of WSWPE was conducted by its repeated oral administration to Sprague–Dawley rats of either sex for a total of 28 days, at the doses of 100, 300 and 1000 mg/kg/day, according to OECD test guideline 407, in compliance with OECD Principles of GLP. Besides, the study design also comprised of a recovery arm in which both male and female rats were administered with the vehicle and the high dose of WSWPE for 28 days following which the administration of the extract was discontinued and rats were observed for reversal, persistence or delayed occurrence of any adverse outcomes for an additional 14 days. In the present study, as compared to the vehicle-treated groups, WSWPE did not reveal any clinically significant alterations in the evaluated parameters. Accordingly, founded on the aforesaid premise, the NOAEL for WSWPE was determined to be 1000 mg/kg/day in both male and female rats. Consequently, the results provision for further non-clinical safety evaluation for WSWPE in non-rodents, and for longer duration in rodents. Additionally, this study also paves the way for the detailed investigation of *Withania somnifera* Whole Plant Extract under clinical settings.

### Supplementary Information

Supplementary Legends.

Supplementary Figure S1.

Supplementary Tables.

## Data Availability

The data generated for the present study are available from the corresponding author on reasonable request.
