# Author Correction: *Withania somnifera* (L.) Dunal whole-plant extract demonstrates acceptable non-clinical safety in rat 28-day subacute toxicity evaluation under GLP-compliance

**DOI:** 10.1038/s41598-024-61198-w

**Published:** 2024-05-10

**Authors:** Acharya Balkrishna, Sandeep Sinha, Jyotish Srivastava, Anurag Varshney

**Affiliations:** 1 Drug Discovery and Development Division, Patanjali Research Institute, NH-58, Roorkee-Haridwar Road, Haridwar, Uttarakhand 249 405 India; 2 Department of Allied and Applied Sciences, University of Patanjali, NH-58, Haridwar, Uttarakhand 249405 India; 3 Patanjali UK Trust, Glasgow, UK; 4 Special Centre for Systems Medicine, Jawaharlal Nehru University, New Delhi, 110067 India

Correction to:* Scientific Reports* 10.1038/s41598-022-14944-x, published online 30 June 2022

The original version of this Article contained errors. Due to errors during figure assembly, in Figure 3 the panel “Heart, 1000 mg/kg/day, Females” was partially overlapping with the panel “Heart, control, Females” and the panel “Bone Marrow, control, Females” was partially overlapping with the panel “Bone Marrow, 1000 mg/kg/day, Males”.

The original Figure 3 appears below as Figure 1.

**Figure 1:**
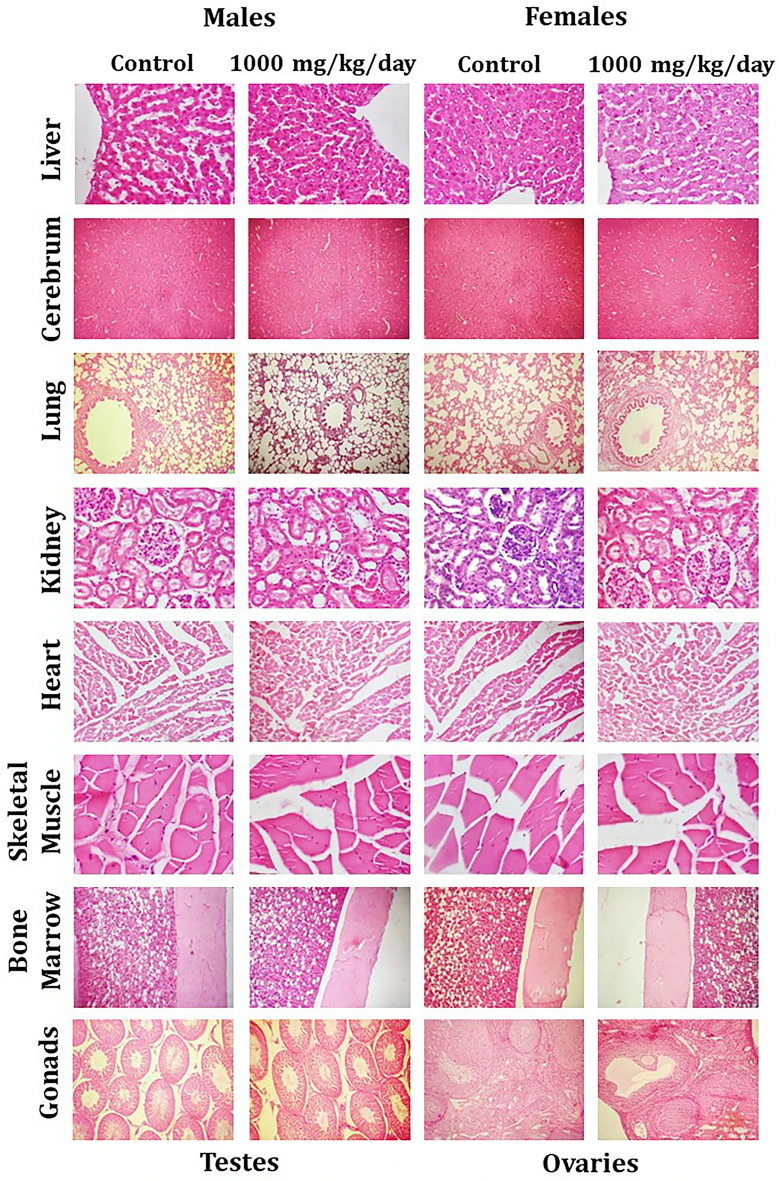
Representative histological photomicrographs of selected organs of rats that orally received either the vehicle (0.5% methylcellulose) or the high dose (1000 mg/kg/day) of WSWPE for a total duration of 28 consecutive days, in this non-clinical safety study. Tissue sections have been stained with Haematoxylin and Eosin. Following organs have been processed for imaging: Cerebrum, Lung, Bone Marrow (× 100); Liver, Kidney, Heart, Skeletal Muscle, Testis, Ovary (× 400).

In addition, in Figure 4 the panel “Adrenal, 1000 mg/kg/day, Females” was partially overlapping with the panel “Adrenal, control, Males”, the panel “Trachea, 1000 mg/kg/day, Females” was partially overlapping with the panel “Trachea, control, Males”, the panel “Pituitary, 1000 mg/kg/day, Females” was partially overlapping with the panel “Pituitary, control, Males”, and the panel “Thyroid, control, Females” was partially overlapping with the panel “Thyroid, control, Males”.

The original Figure 4 appears below as Figure 2.

**Figure 2:**
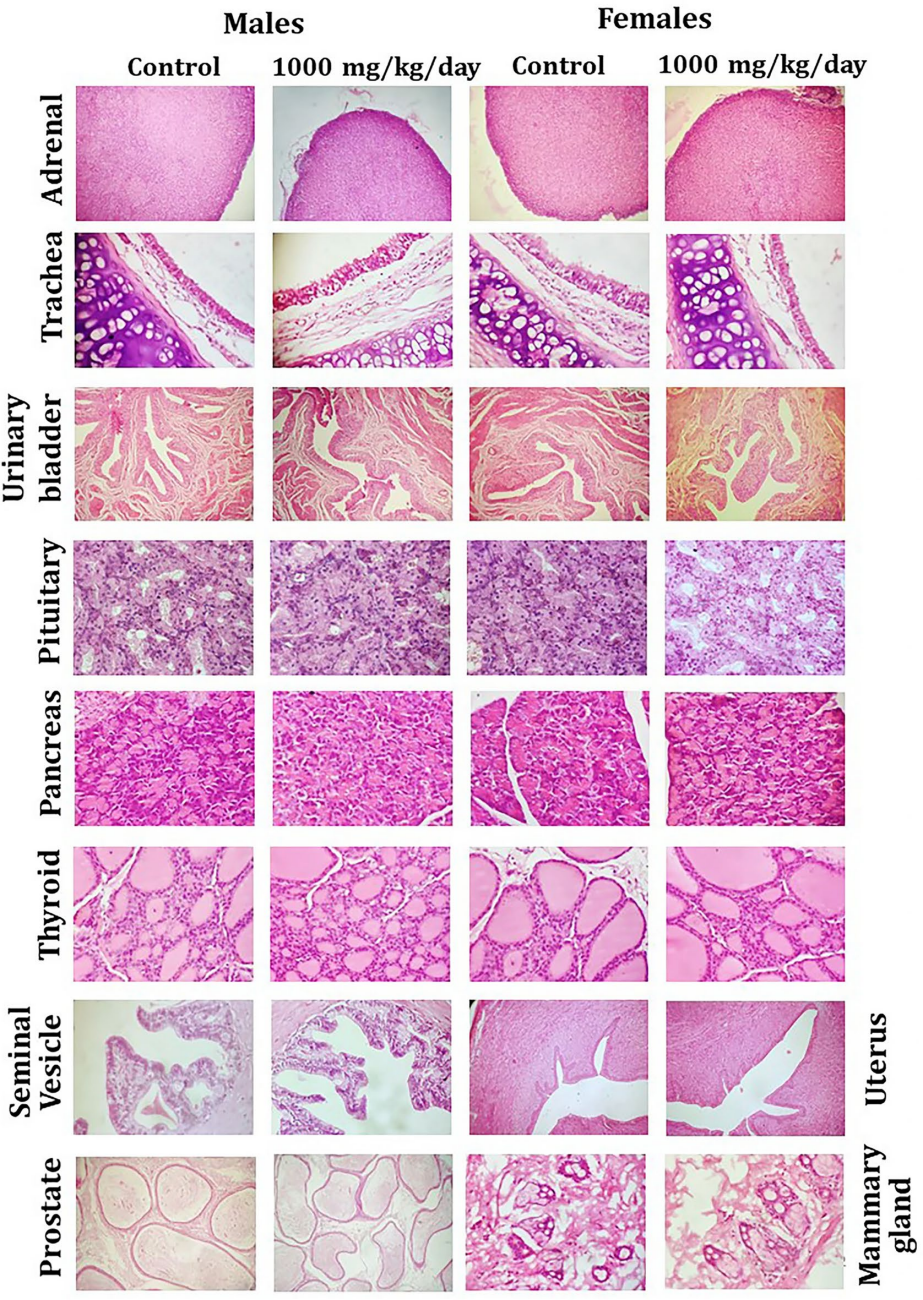
Representative histological photomicrographs of selected organs of rats that orally received either the vehicle (0.5% methylcellulose) or the high dose (1000 mg/kg/day) of WSWPE for a total duration of 28 consecutive days, in this non-clinical safety study. Tissue sections have been stained with Haematoxylin and Eosin. Adrenal gland (× 40); Trachea, Urinary Bladder, Uterus (× 100); Pituitary, Pancreas, Thyroid, Seminal Vesicle, Prostate, Mammary Gland (× 400).

The original Article has been corrected.

