# Supplementary material for: Withania somnifera (L.) Dunal whole-plant extract demonstrates acceptable non-clinical safety in rat 28-day subacute toxicity evaluation under GLP-compliance
Source: Sci Rep. 2022 Jun 30;12:11047. doi: 10.1038/s41598-022-14944-x (PMC9246939; doi:10.1038/s41598-022-14944-x)
Supplement: Supplementary file 1 — Supplementary Legends. [file 41598_2022_14944_MOESM1_ESM.docx]

**SUPPLEMENTARY FIGURE LEGEND**

**Supplementary Figure S1.** Study design of the non-clinical safety assessment of WSWPE in Sprague-Dawley rats. The study design comprised of two arms: 28-day treatment and the 14-day recovery. The study groups in both the arms received either the vehicle (0.5% methylcellulose) or WSWPE by oral route for 28-consecutive days. The animal groups in the recovery arm were additionally observed for a treatment-free period of 14 days. The study observations included monitoring the animals for mortality, morbidity, aberrant clinical signs, ophthalmological examination, detailed clinical observations, alterations in hematological, coagulation, clinical chemistry and qualitative urinalysis parameters. The relative organ weights with respect to the fasting body weights of the animals and any gross or histological changes in the harvested organs were additionally studied.
