# Supplementary material for: Withania somnifera (L.) Dunal whole-plant extract demonstrates acceptable non-clinical safety in rat 28-day subacute toxicity evaluation under GLP-compliance
Source: Sci Rep. 2022 Jun 30;12:11047. doi: 10.1038/s41598-022-14944-x (PMC9246939; doi:10.1038/s41598-022-14944-x)
Supplement: Supplementary file 3 — Supplementary Tables. [file 41598_2022_14944_MOESM3_ESM.docx]

| **S. No.** | **Compound Name** | **Concentration (mg/ml) at initial stage** | **Concentration (mg/ml) after 24 hours at room temperature** |
| --- | --- | --- | --- |
| 1. | Withanoside IV | 0.122 | 0.106 |
| 2. | Withaferin A | 0.257 | 0.211 |
| 3. | 12-Deoxywithastramonolide | 0.343 | 0.283 |
| 4. | Withanolide A | 0.048 | 0.036 |
| 5. | Withanone | 0.006 | 0.004 |
| 6. | Withanolide B | 0.005 | 0.004 |
| 7. | Withanoside V & VI | 0.104 | 0.091 |
|  | Total Withanolides | 0.885 | 0.735 |

**SUPPLEMENTARY TABLE S1.** Composition and concentration of the phytoconstituents in a 100 mg/ml suspension of WSWPE in 0.5% methylcellulose.

| **Parameter** | **Male (N = 5)** | | | | | | **Female (N = 5)** | | | | | | |
| --- | --- | --- | --- | --- | --- | --- | --- | --- | --- | --- | --- | --- | --- |
|  | **28 days treatment (mg/kg/day)** | | | | **14 days recovery (mg/kg/day)** | | **28 days treatment (mg/kg/day)** | | | | | **14 days recovery (mg/kg/day)** | |
|  | **G1 (0)** | **G2 (100)** | **G3 (300)** | **G4 (1000)** | **G1R (0)** | **G4R (1000)** | **G1 (0)** | **G2 (100)** | **G3 (300)** | **G4 (1000)** | **G1R (0)** | | **G4R (1000)** |
| Mortality | 0/5 | 0/5 | 0/5 | 0/5 | 0/5 | 0/5 | 0/5 | 0/5 | 0/5 | 0/5 | 0/5 | | 0/5 |
| Survival | 5/5 | 5/5 | 5/5 | 5/5 | 5/5 | 5/5 | 5/5 | 5/5 | 5/5 | 5/5 | 5/5 | | 5/5 |

**SUPPLEMENTARY TABLE S2.** Observation for mortality in male and female rats. N, Number of animals/Group.

| **Administration Days** | **28 days treatment (mg/kg/day)** | | | | **14 days recovery (mg/kg/day** | |
| --- | --- | --- | --- | --- | --- | --- |
|  | **G1 (0)** | **G2 (100)** | **G3 (300)** | **G4 (1000)** | **G1R (0)** | **G4R (1000)** |
| **Males** | | | | | | |
| 1 | NAD (5/5) | NAD (5/5) | NAD (5/5) | NAD (5/5) | NAD (5/5) | NAD (5/5) |
| 8 | NAD (5/5) | NAD (5/5) | NAD (5/5) | NAD (5/5) | NAD (5/5) | NAD (5/5) |
| 15 | NAD (5/5) | NAD (5/5) | NAD (5/5) | NAD (5/5) | NAD (5/5) | NAD (5/5) |
| 22 | NAD (5/5) | NAD (5/5) | NAD (5/5) | NAD (5/5) | NAD (5/5) | NAD (5/5) |
| 28 | NAD (5/5) | NAD (5/5) | NAD (5/5) | NAD (5/5) | NAD (5/5) | NAD (5/5) |
| 35 | NA | NA | NA | NA | NAD (5/5) | NAD (5/5) |
| 42 | NA | NA | NA | NA | NAD (5/5) | NAD (5/5) |
| **Females** | | | | | | |
| 1 | NAD (5/5) | NAD (5/5) | NAD (5/5) | NAD (5/5) | NAD (5/5) | NAD (5/5) |
| 8 | NAD (5/5) | NAD (5/5) | NAD (5/5) | NAD (5/5) | NAD (5/5) | NAD (5/5) |
| 15 | NAD (5/5) | NAD (5/5) | NAD (5/5) | NAD (5/5) | NAD (5/5) | NAD (5/5) |
| 22 | NAD (5/5) | NAD (5/5) | NAD (5/5) | NAD (5/5) | NAD (5/5) | NAD (5/5) |
| 28 | NAD (5/5) | NAD (5/5) | NAD (5/5) | NAD (5/5) | NAD (5/5) | NAD (5/5) |
| 35 | NA | NA | NA | NA | NAD (5/5) | NAD (5/5) |
| 42 | NA | NA | NA | NA | NAD (5/5) | NAD (5/5) |

**SUPPLEMENTARY TABLE S3.** Detailed clinical observations of rats. NA, not applicable; NAD, no abnormality detected.

| **Day** | **Dose (mg/kg/day)** | | | |
| --- | --- | --- | --- | --- |
|  | **Male (N=5)** | | **Female (N=5)** | |
|  | **G1 (0)** | **G4 (1000)** | **G1 (0)** | **G4 (1000)** |
| 28 | NAD (5/5) | NAD (5/5) | NAD (5/5) | NAD (5/5) |

**SUPPLEMENTARY TABLE S4.** Ophthalmoscopic examination of male and female rats. NAD, no abnormality detected.

| **Sex/Organ/Finding** | **28 days treatment (mg/kg/day)** | | | | **14 days recovery (mg/kg/day)** | |
| --- | --- | --- | --- | --- | --- | --- |
|  | **G1 (0)** | **G2 (100)** | **G3 (300)** | **G4 (1000)** | **G1R (0)** | **G4R (1000)** |
| *Males* |  |  |  |  |  |  |
| NAD | 5/5 | 5/5 | 5/5 | 5/5 | 5/5 | 5/5 |
| *Females* |  |  |  |  |  |  |
| *NAD* | 5/5 | 5/5 | 5/5 | 5/5 | 5/5 | 5/5 |

**SUPPLEMENTARY TABLE S5.** Gross Pathology Observations at Necropsy**.** NAD, no abnormality detected.

| **Organ/Finding/Severity** | **Males** | | **Females** | |
| --- | --- | --- | --- | --- |
|  | **G1 (0)** | **G4 (1000)** | **G1 (0)** | **G4 (1000)** |
| **Lungs** |  |  |  | |
| *Peribronchial MNC infiltration* |  |  |  |  |
| Minimal focal | 1/5 | 2/5 | 1/5 | 1/5 |
| **Liver** |  |  |  |  |
| *Degenerative changes along with MNC*  *infiltration* |  |  |  |  |
| Minimal focal | 1/5 | 2/5 | 2/5 | 1/5 |
| **Kidney** |  |  |  |  |
| *Tubular degeneration and MNC infiltration* |  |  |  |  |
| Minimal focal | 2/5 | 1/5 | 1/5 | 2/5 |
| **Heart** |  |  |  |  |
| *Congestion* |  |  |  |  |
| Minimal focal | 1/5 | 0/5 | 1/5 | 1/5 |

**SUPPLEMENTARY TABLE S6.** Histopathological observations in rats treated with vehicle and the high dose

of WSWPE.
